# Transcatheter Arterial Embolization for Bleeding Related to Pelvic Trauma: Comparison of Technical and Clinical Results between Hemodynamically Stable and Unstable Patients

**DOI:** 10.3390/tomography9050133

**Published:** 2023-09-01

**Authors:** Roberto Minici, Michele Mercurio, Giuseppe Guzzardi, Massimo Venturini, Federico Fontana, Luca Brunese, Pasquale Guerriero, Raffaele Serra, Filippo Piacentino, Marco Spinetta, Lorenzo Zappia, Davide Costa, Andrea Coppola, MGJR Research Team, Olimpio Galasso, Domenico Laganà

**Affiliations:** 1 Radiology Unit, Dulbecco University Hospital, 88100 Catanzaro, Italy; zappial@gmail.com (L.Z.); domenico.lagana@unicz.it (D.L.); 2 Department of Orthopaedic and Trauma Surgery, Magna Græcia University, Dulbecco University Hospital, 88100 Catanzaro, Italy; mercuriomi@gmail.com (M.M.); galasso@unicz.it (O.G.); 3 Radiology Unit, Maggiore della Carità University Hospital, 28100 Novara, Italy; giuguzzardi@gmail.com (G.G.); marcospinetta90@gmail.com (M.S.); 4 Diagnostic and Interventional Radiology Unit, ASST Settelaghi, Insubria University, 21100 Varese, Italy; massimo.venturini@uninsubria.it (M.V.); federico.fontana@uninsubria.it (F.F.); filippo.piacentino@asst-settelaghi.it (F.P.); andrea.coppola@asst-settelaghi.it (A.C.); 5 Department of Medicine and Health Sciences, University of Molise, 86100 Campobasso, Italy; luca.brunese@unimol.it (L.B.); pasqualeguerriero@gmail.com (P.G.); 6 Vascular Surgery Unit, Department of Medical and Surgical Sciences, Magna Graecia University of Catanzaro, Dulbecco University Hospital, 88100 Catanzaro, Italy; rserra@unicz.it; 7 Department of Law, Economics and Sociology, Magna Graecia University of Catanzaro, 88100 Catanzaro, Italy; davide.costa@unicz.it; 8 Magna Graecia Junior Radiologists Research Team, 88100 Catanzaro, Italy; radiologyumg@gmail.com; 9 Department of Experimental and Clinical Medicine, Magna Graecia University of Catanzaro, 88100 Catanzaro, Italy

**Keywords:** angioembolization, trauma, pelvic injury, hemodynamic instability, transcatheter arterial embolization, bleeding, hemorrhage, endovascular, percutaneous, prophylactic embolization

## Abstract

Background: Endovascular intervention is now the primary line of therapy for arterial injury brought on by pelvic trauma since it can significantly reduce considerable morbidity associated with surgery and can swiftly access and control bleeding sites. Despite international guidelines and widespread awareness of the role of angioembolization in clinical practice, robust evidence comparing the outcomes of angioembolization in hemodynamically stable and unstable patients is still lacking. This study aims to directly compare the outcomes of angioembolization for the treatment of pelvic traumatic arterial injury in patients with hemodynamic stability vs. hemodynamic instability. Methods: In our multicenter retrospective investigation, we analyzed data from consecutive patients who underwent, from January 2020 to May 2023, angioembolization for traumatic pelvic arterial injury. Results: In total, 116 angioembolizations were performed. Gelatin sponges (56.9%) and coils (25.9%) were the most widely used embolic agents. The technical and clinical success rates were 100% and 91.4%, respectively. No statistically significant differences were observed between the two groups in terms of technical success, clinical success, procedure-related complication rate, or 30-day bleeding-related mortality. Conclusions: Angioembolization is an effective and safe option for the management of traumatic pelvic arterial lesions even in hemodynamically unstable patients, despite technical variations such as greater use of prophylactic angioembolization.

## 1. Introduction

In the United States, trauma is the leading cause of mortality and disability in the younger population [1]. Trauma has also a significant financial impact, accounting for 12% of all healthcare expenses worldwide [2]. With a high mortality rate (between 5 and 50%), pelvic fractures are among the most serious skeletal injuries [3]. Endovascular intervention is now the primary line of therapy for arterial injury brought on by pelvic trauma since it can significantly reduce considerable morbidity associated with surgery and can swiftly access and control bleeding sites [4,5]. 

Roughly up to 10% of all patients with a pelvic injury experience arterial bleeding, although a venous or bony origin accounts for the majority [6,7,8,9,10]. The frequency of arterial injury rises up to 60% in patients with unstable pelvic fractures [6,7,11]. Although large blood losses may also occur in venous bleeding, hemodynamic instability is seen more frequently for arterial lesions [10]. In patients with pelvic injuries who do not respond to fluid resuscitation or blood transfusions, more than 70% have an arterial cause of bleeding [6]. Interestingly, pelvic trauma patients with hemodynamic instability experience a mortality rate of up to 56% [6,7,12].

Athanasoulis et al. first described the use of angiography (AG) to identify the bleeding site in patients with pelvic trauma [13], while Margolies et al. published, in 1972, the first report on angioembolization (AE) in patients with pelvic trauma using autologous clots [14]. Currently, the World Society of Emergency Surgery management guidelines for pelvic trauma reserve a pivotal role for transcatheter arterial embolization (TAE) in both hemodynamically stable and unstable patients, especially if the surgical option is not deemed appropriate [5]. Of note, a dedicated interventional radiology team is required on standby or on call, with around-the-clock access to a catheterization lab [15,16,17].

Despite international guidelines and widespread awareness of the role of AE in clinical practice [5,8,18,19,20,21,22,23], evidence directly comparing the outcomes of AE in hemodynamically stable and unstable patients is still lacking. Indeed, it is useful to note that the hemodynamically unstable patient is a critically ill patient with high mortality and that the time-sensitive scenario influences the AE technique and the choice of the embolizing agent [4,24,25]. Therefore, it would be desirable to be aware of any AE procedural or outcome differences between hemodynamically stable and unstable patients. The aim of the study was to directly compare the outcomes of angioembolization for the treatment of pelvic traumatic arterial injury in patients with hemodynamic stability vs. hemodynamic instability.

## 2. Materials and Methods

### 2.1. Study Design

**Figure 1:**
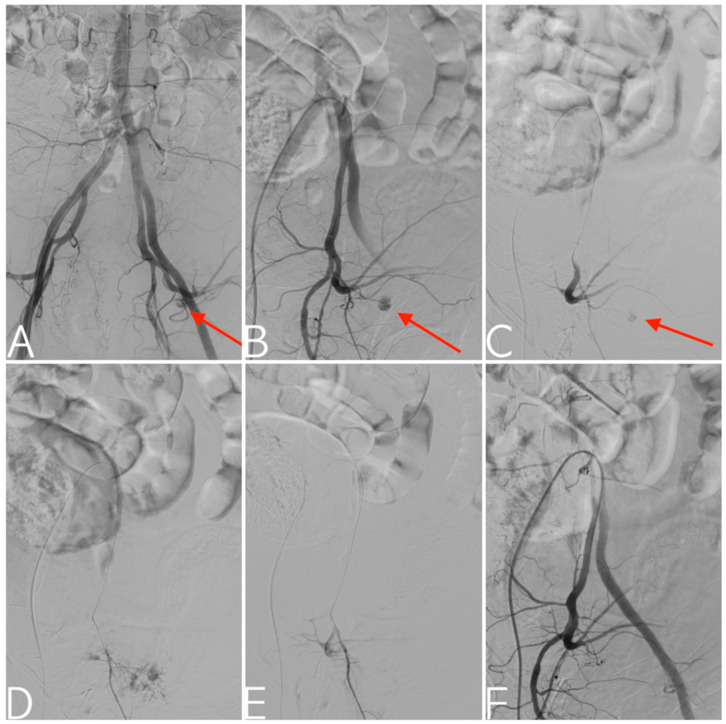
Fall off a horse. Aortoiliac digital subtraction angiography depicting arterial injury (arrows) to the posterior division of the left internal iliac artery (**A**), confirmed by the selective catheterization of the internal iliac artery (**B**) and its posterior division (**C**). Given the young age of the patient, an attempt was made to superselectively catheterize the small branch of the superior gluteal artery responsible for the bleeding (**D**). Targeted embolization using a gelatin sponge (**E**), with technical success and preserved patency of the main trunk of the superior gluteal artery documented in the final angiogram (**F**).

**Figure 2:**
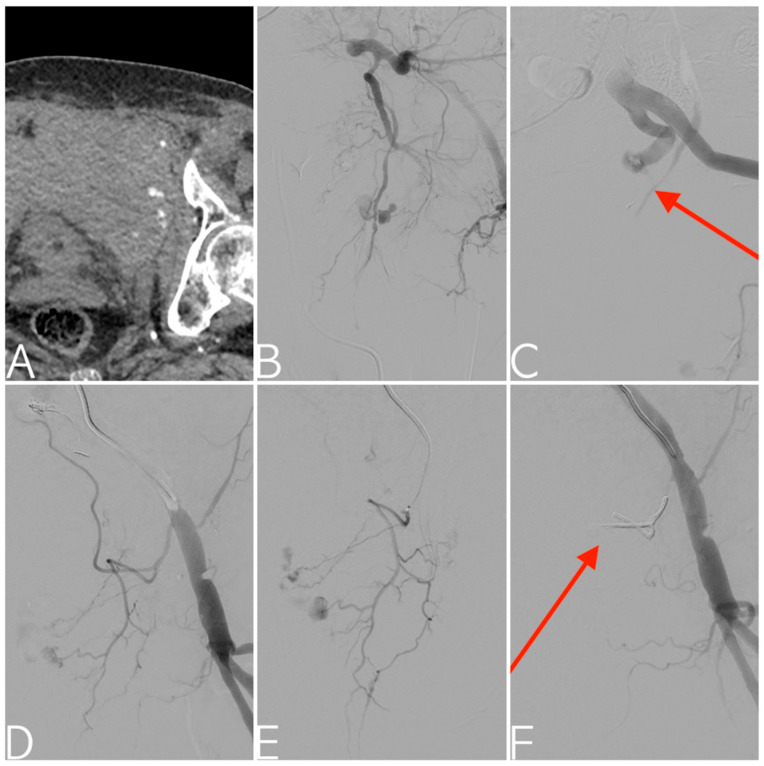
CT angiography scan of motorcycle accident patient showing an open-book pelvic fracture with active contrast extravasation into a Retzius space hematoma (**A**). Bleeding from the left internal pudendal artery confirmed with selective digital subtraction angiography (**B**). The ensuing vasospasm makes both distal selective catheterization and the correct proximal release of coils (arrow) in the anterior division impossible; therefore, prophylactic embolization is performed with a gelatin sponge, also in consideration of a worsening of the patient’s hemodynamic status (**C**). Digital subtraction angiography of the external iliac artery showing active bleeding from the external pudendal artery (**D**), confirmed with superselective catheterization (**E**). Final angiogram demonstrating effective embolization with EVOH copolymer cast (arrow) (**F**).

**Figure 3:**
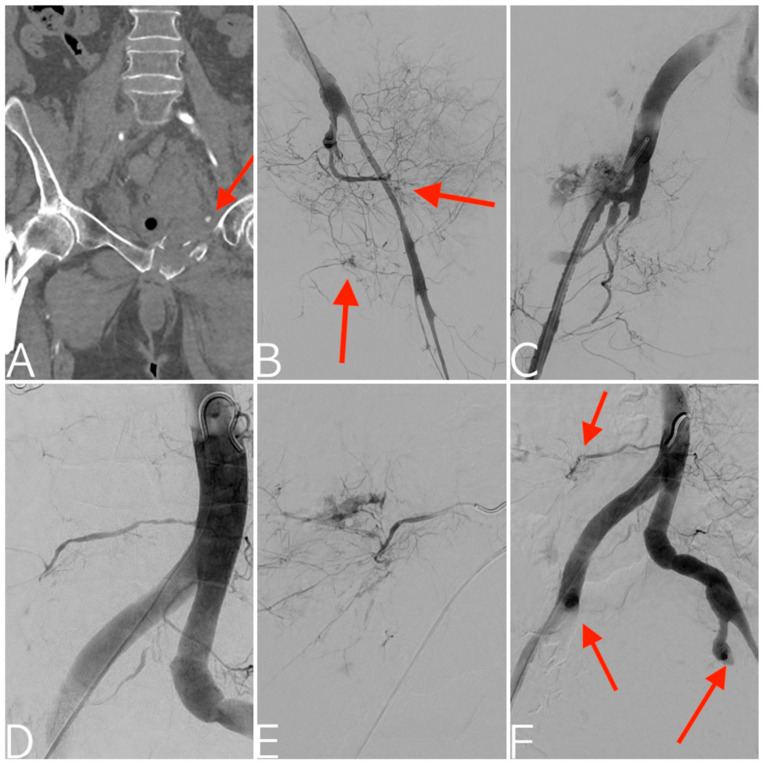
Pedestrian versus motorcycle accident. CT angiography with multiplanar reconstruction in the coronal plane depicting right femoral neck and left superior pubic ramus fractures with active contrast extravasation (arrow) (**A**). Digital subtraction angiography documenting severe vasospasm of the left internal iliac artery, along with active bleeding from both anterior and posterior divisions (**B**). Subsequent state of hemodynamic instability with consequent proximal prophylactic embolization using a gelatin sponge. Contralateral digital subtraction angiography depicting vasospasm of the right internal iliac artery together with severe arterial injury of the posterior division (**C**). This is followed by embolization using an EVOH copolymer. Aortogram showing right fifth lumbar artery bleeding (**D**), confirmed with superselective catheterization (**E**) and successfully managed with coils embolization. Final aortogram depicting the bilateral proximal embolization of both internal iliac arteries and right fifth lumbar artery embolization (arrows) (**F**).

**Figure 4:**
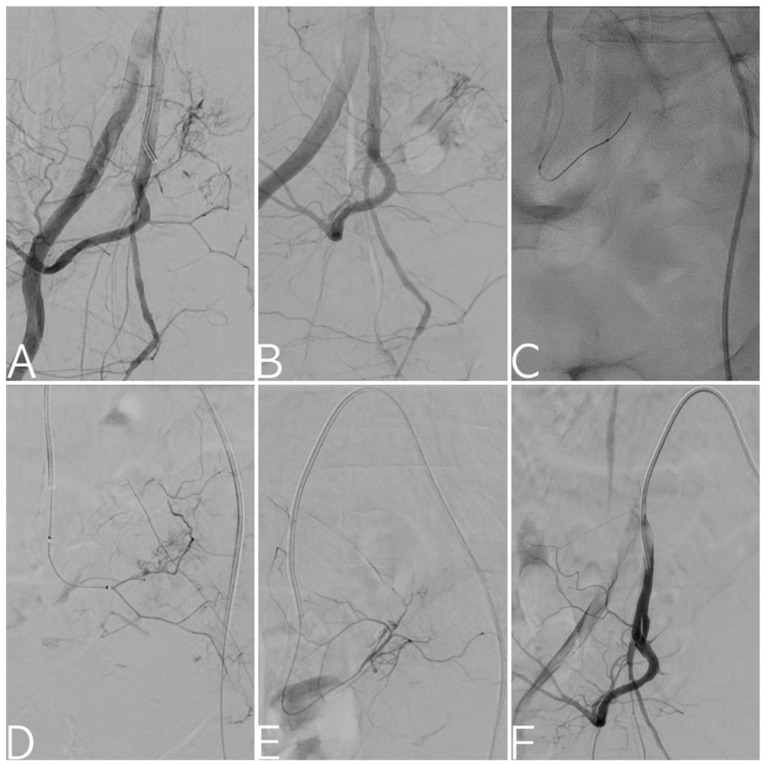
Selective digital subtraction angiography of the right internal iliac artery: bleeding from the lateral sacral arteries noted (**A**,**B**). Fluoroscopy image depicting superselective catheterization of the target vessel with 2.4 Fr microcatheter and 0.018” guidewire (**C**). Angiograms confirming bleeding from the lateral sacral arteries (**D**,**E**), treated via targeted embolization with a gelatin sponge. Final angiogram demonstrating technical success with exclusion of arterial injury from circulation (**F**).

In our multicenter (Dulbecco University Hospital, Catanzaro, Italy; Circolo Hospital, Varese, Italy; Maggiore della Carità University Hospital, Novara, Italy; Mater Domini University Hospital, Catanzaro, Italy; Pugliese-Ciaccio Hospital, Catanzaro, Italy) retrospective cohort evaluation, we analyzed data from consecutive patients who underwent, from January 2020 to May 2023, AE for traumatic pelvic arterial injury (Figure 1, Figure 2, Figure 3 and Figure 4). Patients who met the following inclusion criteria were enrolled: (I) transcatheter arterial embolization of traumatic pelvic arterial injury, performed according to the indications stated in Section 2.2; (II) 18 years of age or older; (III) evaluation in a multidisciplinary team composed of surgeons, interventional radiologists, and anesthesiologists. The exclusion criteria were (I) pregnant females; (II) according to SIR recommendations, a platelet count below 20,000/μL and a refusal to receive a transfusion of blood component [26]; (III) international normalized ratio (INR) not compatible with femoral (>1.8) or radial (>2.2) artery access for low bleeding risk operations requiring arterial access [26]; (IV) known hypersensitivity to suitable embolic agents; (V) access to an emergency room of another hospital and subsequent transfer.

The study was retrospective; thus, no permission from an ethical committee was required. The research was conducted in compliance with the principles set forth in the Declaration of Helsinki. Before endovascular procedures, each patient provided written informed consent.

### 2.2. Treatment 

Indications for AG included:

1. Patients with hemodynamic instability who underwent temporary pelvic stabilization, the start of aggressive volume resuscitation, and exclusion of extra-pelvic blood loss [5];
2. Patients with extra-pelvic blood loss, persistent hemodynamic instability, and ongoing bleeding even after surgery and preperitoneal pelvic packing (PPP), or simultaneously to surgery and PPP if a hybrid operating room was available [4,5,18];
3. Patients with previous hemodynamic instability and extra-pelvic blood loss, undergoing surgery and PPP resulting in hemodynamic stability; subsequent CT scan with evidence of arterial bleeding [4,18];
4. Patients who already underwent pelvic AG with or without AE, with persistent signs of ongoing bleeding and exclusion of extra-pelvic blood loss [5];
5. Elderly patients (>55 years), regardless of hemodynamic status [4,5,27];
6. Hemodynamically stable patients with unstable pelvic fractures and direct and/or indirect CT signs of arterial bleeding [4,5,28,29,30];
7. Hemodynamically stable patients with stable pelvic fractures or direct and/or indirect CT signs of arterial bleeding, based on the presence of clinical signs of active bleeding [4,31];
8. Hemodynamically stable patients with stable/unstable pelvic fractures or unremarkable CT scans but still clinical signs of significant ongoing bleeding. The rationale was to differentiate venous or bone bleeding from arterial bleeding that may be absent or unidentifiable at the time of CT. The clinical significance of the ongoing bleeding may justify the continuation of the imaging workup with AG, as an absence of blush at CT does not always exclude active pelvic bleeding at AG [4,32,33]. It is worth noting that sacroiliac joint disruption and female gender were proven to be reliable predictors of patients who would benefit from pelvic AG [34].

Indications for AE included a targeted selective AE, guided by AG finding active extravasation or indirect signs of arterial injury (e.g., pseudoaneurysm, arteriovenous fistula, vascular cut-off sign, vasospasm); a prophylactic AE consisting of rapid nonselective proximal occlusion of the posterior and/or anterior division of the internal iliac artery; or, in some particular conditions of multiple vascular lesions and hemodynamic instability, in the bilateral occlusion of both internal iliac arteries [4,5].

Prophylactic (or nonselective proximal) embolization is justified in some clinical scenarios: (1) multiple bleeding vessels and/or technically not feasible targeted embolization; (2) hemodynamically unstable patient, exclusion of other sources of extra-pelvic bleeding, no evidence of active extravasation upon AG but identification of the suspected target vessel based on the anatomical location of the fractures identified on a plain radiograph or CT (blind embolization); (3) hemodynamically unstable patients with poor clinical conditions, thus requiring very rapid embolization [4,5,25].

Diagnostic workup and treatment strategies followed the World Society of Emergency Surgery (WSES) guidelines for pelvic trauma [5], together with some specific clinical scenarios defined by Renzulli et al. [4]. In patients with hemodynamic instability, chest and pelvic X-rays and extended focused assessment for sonographic evaluation of trauma patients (E-FAST) were performed to exclude extra-pelvic blood loss in the thorax and in the abdomen. Salim et al. demonstrated that the duration of hypotension positively predicts contrast extravasation and the need for therapeutic AE, thus explaining why hemodynamically unstable patients should empirically undergo angiography, without prior CT examination [34]. In hemodynamically stable patients, the gold-standard imaging work-up is represented by CT angiography (CTA). The endovascular procedure was performed in dedicated catheterization labs by an experienced interventional radiologist (at least 5 years of experience), with a standard retrograde approach via the contralateral common femoral artery. Diagnostic AG has always preceded the AE. In cases of positive CT and negative AG for bleeding, the AG was declared to be over only after selective catheterization of the artery suspected of bleeding on the CT, identified via direct/indirect signs of bleeding and/or the anatomical location of the fracture [4]. The operator’s preferences determined the embolic agent to use. The instructions for use were followed for the preparation of each embolic agent and subsequently applied under fluoroscopic guidance. After applying N-butyl cyanoacrylate (NBCA) or a non-adhesive liquid embolic agent (NALEA), the microcatheter was never used again. Given the presence of anastomoses such as those between the anterior and posterior division of the internal iliac artery and between the right and left internal iliac arteries, targeted AEs of proximal vascular lesions were performed with sandwich embolization of afferent and efferent vessels, thus preventing backdoor bleeding. Assessments of technical success and non-target embolization were performed via postembolization AG, with selective AGs of both internal iliac arteries and an aortoiliac angiography. All patients underwent clinical evaluation and possible follow-up imaging before hospital discharge and 1 month after TAE.

### 2.3. Outcomes and Definitions

The primary efficacy endpoint was to report the clinical success rate in hemodynamically stable and unstable patients. The secondary efficacy endpoint was to investigate the difference in 30-day bleeding-related mortality between the 2 groups. The difference in procedure-related complication rate between the 2 groups was considered the primary safety endpoint. 

According to advanced trauma life support (ATLS), hemodynamic instability is defined as blood pressure < 90 mmHg and heart rate > 120 bpm, with evidence of skin vasoconstriction, an altered level of consciousness, and/or shortness of breath. Based on the patient’s response to volume resuscitation, responders or transient responders are considered hemodynamically stable patients [2]. These specified criteria were used to separate the population into two distinct groups: one characterized by stable hemodynamics and the other exhibiting unstable hemodynamics. The mechanical stability of pelvic ring injuries is evaluated according to the Young–Burgess classification [35]. Mechanical and hemodynamic stability allows patients to be grouped into 4 categories according to the WSES pelvic trauma classification [5]. Contrast blush is a direct sign of bleeding at CT, as it is a reliable predictor of active arterial bleeding at AG [31,36]. Indirect signs of ongoing/recent arterial bleeding (but not necessarily bleeding during the CT exam [4]) at CT included large-volume pelvic hematoma, pseudoaneurysm, arteriovenous fistula, vasospasm, or a vascular cut-off sign (i.e., amputated/truncated vessel implying dissection/thrombosis, transection, or vasospasm of the involved artery) [4,6,37,38,39,40,41]. Interestingly, a pelvic hematoma ≥ 500 cm^3^ is a strong predictor of arterial injury even in the absence of a visible contrast blush [32]. Selective (distal) embolization is defined by the occlusion of the bleeding vessel or its parent artery if the bleeding vessel is one of its small distal branches. Nonselective (proximal) embolization is defined by occlusion of the bleeding vessel and vessels other than the bleeding vessel or, if the bleeding vessel is very small and distal, by occlusion of arterial branches that do not share the same parent artery of the bleeding vessel (i.e., bleeding of small distal branches of the internal pudendal artery: occlusion of the bleeding vessel or internal pudendal artery configures distal selective embolization, while occlusion of the anterior division of the internal iliac artery or the entire internal iliac artery configures proximal nonselective embolization). An INR greater than 1.5 and/or a partial thromboplastin time longer than 45 s and/or a platelet count less than 80,000/mm^3^ defined the coagulopathy condition [42]. The Injury Severity Score (ISS) was used to assess trauma severity, thus taking into account injuries involving six body regions [43]. Time-to-embolization (TTE) was calculated as the time elapsed between access to the emergency room and AE. SIR reporting standards were used [44]. The procedure-related complication rate includes vascular access site complication (VASC) rate. Procedure-related complications were classified according to the 2017 SIR classification [45], the 2003 SIR classification [46], and the CIRSE classification [47].

### 2.4. Statistical Analysis

Data were maintained in an Excel spreadsheet (Microsoft Inc., Redmond, WA, USA), and the statistical analyses were performed on an intention-to-treat basis using the SPSS software (SPSS, v. 22 for Windows; SPSS Inc., Chicago IL, USA) and the R/R Studio software. All randomized patients undergoing at least one embolization procedure comprised the modified intention-to-treat population used for the analyses [48,49]. The normality assumption of the data was verified using the Kolmogorov–Smirnov and Shapiro–Wilk tests. Frequency (% value) is the presentation format for categorical data [50]. Data with a continuous normal distribution are shown as mean ± standard deviation. Data that are continuous but not normally distributed are shown as the median (first to third quartile) [51,52]. An unpaired Student’s *t*-test was used to assess statistical differences for continuous normally distributed data, while categorical and continuous not normally distributed data were assessed using the Chi-squared/Fisher’s exact tests and the Mann–Whitney test, respectively [53,54,55]. A *p*-value of <0.05 was considered statistically significant for the aforementioned tests.

## 3. Results

During the study interval, 116 patients underwent angioembolization for post-traumatic pelvic arterial injury. In 36 (31.0%) patients, a coagulopathy condition was noted. The mean baseline value of serum hemoglobin, recorded in the preprocedural phase, was 7.9 (±0.9) g/dL. Most pelvic injuries occurred with a blunt mechanism (87.9%). Stable pelvic fractures accounted for the majority of pelvic ring injuries. In total, 37.9% of the patients were in conditions of hemodynamic instability. The mean Injury Severity Score was 28.8 (±15) points, and extra-pelvic injuries were observed in 78 (67.2%) cases. Details are provided in Table 1.

In total, 116 angioembolizations were performed. Blind embolization was executed in six (5.2%) patients, as no bleeding was noted on X-ray angiography (XA). The most frequent bleeding site was the internal iliac artery and its branches (89.7%). A substantial equal distribution of prophylactic and distal angioembolizations was noted. Gelatin sponges (56.9%) and coils (25.9%) were the most widely used embolic agents. Common femoral arteries accounted for 93.1% of the vascular access sites. In 27 (22.5%) cases, Onyx or Squid was used. The mean volume of iodinated contrast media used during embolizations was 38.1 (±14) mL. The mean volume of the contrast-to-creatinine-clearance ratio was 0.73 (±0.7). The most used vascular access site was the common femoral artery (70%). The mean door-to-groin time, the mean procedure time, and the mean time-to-embolization were 91.2 (±76.2) minutes, 30.2 (±10.9) minutes, and 120.1 (±75.4) minutes, respectively. The procedure data are detailed in Table 2.

The technical and clinical success rates were 100% and 91.4%, respectively. Twenty-two cases of rebleeding required repeat XAs, with evidence in most cases (63.6%) of a different bleeding site from the previous one. During the patients’ hospital stays, the occurrence of trauma-induced coagulopathy (TIC) was noted in 28 (24.1%) cases. Procedure-related complications were observed in 14 (12.1%) patients. Vascular access site complications (VASCs) were recorded in four (3.4%) cases, related to a case of access site hematoma (>5 cm), two pseudoaneurysms, and a case of vascular access site thrombosis. Pseudoaneurysms and access site hematomas were managed conservatively using ultrasound-guided compression and prolonged access site compression, respectively. Vascular access site thrombosis required surgical treatment. Other complications included three abscesses and seven contrast-induced nephropathies. The 30-day bleeding-related mortality and 30-day mortality rates were 12.1% and 17.2%, respectively. Details are provided in Table 3.

Patients were divided into two groups according to hemodynamic status. A trend without statistical significance toward lower baseline serum hemoglobin values was noted in the group with hemodynamic instability (8 ± 0.3 g/dL vs. 7.7 ± 0.8 g/dL; *p* = 0.0869). The rate of prophylactic angioembolization was significantly higher in patients with hemodynamic instability (Group 2) than in patients with hemodynamic stability (Group 1) (90.9% vs. 27%; *p* < 0.0001). A gelatin sponge was used more frequently as an embolic agent in the hemodynamic instability group (86.4% vs. 38.9%; *p* < 0.0001). A longer time-to-embolization was noted in the hemodynamically stable group (139.2 vs. 64.6 min; *p* < 0.0001). No statistically significant differences were observed between the two groups in terms of technical success, clinical success, procedure-related complication rate, 30-day mortality, or 30-day bleeding-related mortality. However, a nonsignificant trend toward higher 30-day mortality can be seen in the hemodynamic instability group (22.7% vs. 13.9%; *p* = 0.3108).

A comparison of data between Group 1 and Group 2 is reported in Table 4.

The patients were divided into two groups according to angioembolization techniques (prophylactic vs. targeted). No statistically significant differences were observed between the two groups in terms of technical success, rebleeding, or procedure-related complication rate. A significant trend toward a higher clinical success rate was noted in the group undergoing prophylactic angioembolization (100% vs. 82.1%; *p* = 0.001).

A brief comparison of efficacy and safety data between prophylactic and targeted angioembolization is reported in Table 5.

## 4. Discussion

In our study, TAE was effective for the management of bleeding related to pelvic trauma. Furthermore, the technical success rate and the clinical success rate were comparable between the hemodynamically stable patients and the hemodynamically unstable patients, indicating that AE is a reliable and effective technique even in critically unstable patients. Increasingly better awareness of the indications for AG/AE, increased knowledge of different embolic agents, and growing experience in some techniques allowed for this result. Previous evidence has demonstrated that the technical success rate ranges between 90 and 100% [8]. The German pelvic injury register showed a 98% technical success rate for AE in pelvic hemorrhage control [39]. Previous reports observed a 74–100% immediate clinical success rate [56,57,58,59]. In their case series on 19 trauma patients with pelvic fractures, Barentsz et al. reported 100% technical success with 74% clinical success [59]. Velmahos et al. highlighted a 93% clinical success rate in a mixed cohort of trauma patients [60]. Although the comparison is hindered by different definitions of technical/clinical success and by populations presenting heterogeneity in AG/AE indications and clinical characteristics, we can state that the efficacy outcomes of our study are consistent with similar reports in the broader field of endovascular embolizations and in the specific field of AE in pelvic trauma patients [8,44,61,62,63,64].

The mortality rate in patients undergoing pelvic AE ranged from 4 to 56%, in agreement with a recent literature review by Wijffels et al. [8]. Older reports recorded a mortality rate of 88.9% because of mean TTE values exceeding 4 h, outdated technology, and poor awareness of AG/AE indications [65]. Metsemakers et al. noted a 20% mortality rate in their report on patients with unstable fractures undergoing TAE because of persistent hemodynamic instability despite adequate fluid resuscitation and emergent surgical fracture stabilization [66]. A high rate of concomitant life-threatening extra-pelvic injuries should be taken into account when evaluating the mortality rate of patients undergoing AE [67], especially in hemodynamically unstable patients, as demonstrated in the current study. Giannoudis et al. observed that 95.2% of non-survivors with pelvic trauma had other extra-pelvic injuries (vs. 68.7% of survivors with pelvic trauma; *p* = 0.01) [68]. Also, Agolini et al. highlighted a higher rate of hemodynamic instability in non-survivors [38]. Furthermore, the results of AE can be jeopardized by the onset of a late trauma-induced coagulopathy (TIC) [69]. We observed a trend toward a higher 30-day mortality rate in the hemodynamic instability group, but we believe that these data must be related to the presence of some confounding factors such as extra-pelvic lesions or the onset of medical conditions such as TIC. This speculation is supported by a very similar 30-day bleeding-related mortality rate between the two groups (hemodynamic stability vs. hemodynamic instability). Thus, the hemodynamically unstable patient with pelvic trauma remains a critically ill patient requiring challenging multidisciplinary management, even after successful AE that resolved to bleed.

A controversial clinical scenario is represented by hemodynamically stable patients, with stable pelvic fractures and a CT finding of contrast blush. If contrast blush is noted on CT angiography, the likelihood of active contrast extravasation at AG is very high [38,56,70]. However, the significant improvement in imaging technology in recent years has led to increased detection of contrast blush on CTs, raising questions about the clinical significance of these findings [71]. In the investigation by Verbeek et al. [28], 53% of patients with pelvic blush did not require any intervention to control pelvic bleeding; other reports have highlighted similar data [30,72]. Therefore, we support the concept that the clinical suspicion of bleeding should guide the decision to perform AG on the hemodynamically stable patient with a stable pelvic fracture and a CT finding of contrast blush [73].

Sometimes the AG does not confirm the bleeding detected in the CTA. It has been speculated that vessel spasms may be responsible for this discrepancy, secondary to the local inflammatory response generated by bleeding and hypotension [4,74]. Therefore, the question arises about possible AE in the case of positive CTA and negative AG for bleeding. In this clinical scenario, we believe it is prudent to support AE only in the event of the patient’s ensuing hemodynamic instability [4]. Future perspectives include prospective observations of patients with hemodynamic stability, positive CTA, negative AG, and denial of AE in order to understand the rate of repeat AGs and the rate of AE at the second AG. In our investigation, we performed six AEs in patients with positive CTA, negative AG, and ensuing hemodynamic instability, with 100% technical and clinical success and no cases of procedure-related complications or rebleeding. A prophylactic AE, which can also be considered blinded, was performed on the anterior or posterior division of the internal iliac artery based on the suspected target vessel found on CTA. Direct visualization of the target vessel on CTA can sometimes be difficult; therefore, it is useful to note that numerous studies have demonstrated that the anatomical location of fractures and hematomas can predict the bleeding vessel, thus guiding AE (e.g., the iliolumbar artery is frequently responsible for bleeding in iliac or sacroiliac joint fractures) [27,75,76].

The use of the anatomical fracture/hematoma location to predict bleeding vessels may also be useful for a hemodynamically stable patient undergoing AG to guide the selective catheterization of the target vessel and reduce time-to-embolization (TTE) in cases of targeted selective AE. In a previous report by Agolini et al., AE within 3 h of arrival at the trauma department was associated with better survival (mortality rate of 14% vs. 75%) [38]. Subsequent evidence is consistent with these data. Tanizaki et al. noted an increased mortality rate in patients undergoing AE more than 60 min after arrival (64% vs. 16%; *p* = 0.04) [77]. Chou et al. demonstrated that TTE positively correlates with the requirement for blood transfusion and intensive care unit (ICU) length of stay (LOS) [76]. In our report, a shorter TTE in the group with hemodynamic instability was noted. Interestingly, the hemodynamic instability group experienced a significantly higher rate of proximal AE, thus fueling speculation about the role of prophylactic AE in time-sensitive scenarios.

The use of prophylactic embolization is another controversial point. Some authors have advocated prophylactic embolization for time-sensitive scenarios such as cases of hemodynamic instability in particularly bad clinical conditions or other associated life-threatening medical conditions [4,25]. Instead, other authors have demonstrated that selective targeted embolization is as rapid as proximal embolization [78]. Another scenario is the hemodynamically unstable patient, without other sources of extra-pelvic bleeding and no evidence of active extravasation on angiography: the pattern of pelvic injury can also be helpful in predicting the site of arterial injury [79]. The main concern of prophylactic iliac artery AE is procedure-related complications, especially if the occlusion is bilateral. According to Travis et al., bilateral internal iliac artery embolization is associated with buttock, thigh, or perineal paresthesia [80]. Another report showed that bilateral AE was performed on every patient with a reported complication [81]. However, other investigations reported no complications overall or no complications associated with bilateral nonselective AE [59,60,77]. Our study supports this speculation, as prophylactic AEs were significantly higher in the group of patients with hemodynamic instability, but the complication rate did not differ significantly. Furthermore, we did not observe significant differences in procedure-related complications or technical success rates between prophylactic and targeted AE. Takahira et al. theorized in their investigation that gluteal muscle necrosis may have been the result of the initial trauma rather than a result of proximal embolization [82]. Therefore, we agree with the previously defended notion that complications have a mixed etiology related to trauma and AE, and the putative and low risk of procedure-related complications is outweighed by the potentially life-saving effect of emergency bilateral prophylactic AE [25,80].

A recent literature review reported a complication rate between 0 and 63% [25]. This heterogeneity could be explained by factors such as the difficult differentiation between procedure-related complications and trauma-related complications; variability in the sample size, which makes it more difficult for rarer complications to occur in investigations with small populations; and different TTEs, as well as indications for AG/AE between the different reports. Complications may involve the vascular access site or be related to the endovascular procedure itself. A wide range of procedure-related complications has been described, including contrast allergy, contrast-induced nephropathy (CIN), gluteal muscle necrosis, nerve lesions and paresthesia, skin necrosis, bladder ischemia, failure in surgical wound healing, femoral head necrosis, deep infections, and abscesses [39,59,60,77,80,81,82,83,84,85,86,87]. In our study, the rate of procedure-related complications, including VASCs, was similar between the hemodynamically stable and unstable patients and comparable to other studies on AEs in pelvic trauma patients [25] and other treatments in the endovascular field [85,88,89,90,91,92,93,94,95,96,97,98,99,100,101]. Finally, some authors have speculated that leaving the distal small vessels patent is critical to preserving sufficient collaterals, thus avoiding significant distal ischemia. In this context, the choice of the best embolic agent and the most adequate embolization technique remains disputed [77,82].

Furthermore, in the group with hemodynamic instability, significantly more frequent use of gelatin sponges was noted. Gelatin sponges are water-soluble temporary embolic agents that are reabsorbed within 2–3 weeks [102]. It is possible to speculate that the group with hemodynamic instability, subjected to a greater number of proximal prophylactic AEs and embolized vessels, received more AEs using gelatin sponges, as operators felt more confident in using a temporary embolic agent in these clinical scenarios [103]. This choice did not result in significant differences in terms of efficacy and safety outcomes between the groups with and without hemodynamic instability. Furthermore, previous evidence has shown that temporary embolic agents are preferred when the hemorrhage involves vital organs, such as the penis, vagina, and ovaries [4,24]. Currently, many embolic agents are available, and the choice depends on the experience of the interventional radiologist and the patient’s clinical setting. One of the factors that may influence the choice of an embolic agent is the presence of coagulopathy [42]. Trauma-induced coagulopathy (TIC) involves abnormal coagulation processes related to trauma. Soon after trauma, TIC is associated with a hypocoagulable state and a tendency for bleeding disorders, while later, TIC is characterized by a hypercoagulable state associated with venous thromboembolism and multiple organ failure [69]. Evaluating the outcomes of different embolic agents in patients with TIC is beyond the scope of this study, but we deem it useful to highlight some speculations. Coagulopathy reduces the efficacy of AE performed with gelatin sponges and coils, as their mechanisms of action are primarily based on clot formation [42,104,105,106,107,108,109]. The use of coils alone is a predictor of rebleeding in patients with coagulopathy [42,110]. Many authors have advocated for the use of non-adhesive liquid embolic agent (NALEA) and n-butyl cyanoacrylate (NBCA) in patients with coagulopathy since their embolization mechanisms do not depend on the thrombus formation but on the mechanical actions of polymerization (NBCA) and solidification (NALEA) [92,109,111]. Interestingly, the fluid-like advancement permits the transportation of liquid embolic agents through narrow arteries, enabling them to reach embolization targets located notably distant from the tip of the microcatheter [112]. Furthermore, opting for liquid embolic agents is advisable when conducting vessel embolization in cases of vessel damage (e.g., aspergilloma, tuberculous, and cancerous lesions) because their deployment mechanism avoids exerting radial force on vessel walls. This stands in contrast to coils and vascular plugs, which can potentially cause ruptures in delicate vessels [113]. Finally, although there is a growing recognition of the effectiveness of PVA particles in embolization procedures, their utilization for pelvic hemorrhages is still supported only by limited evidence [114,115,116].

Reported rates of repeated AGs range from 0% to 23%; our data are in keeping with previous reports [6,9,10,117,118]. The indication for reoperation is represented by the suspected persistence/recurrence of bleeding with the exclusion of other significant sources of bleeding [5]. Some findings have been reported to be reliable predictors of rebleeding, such as hypotension, more than two arterial injuries, baseline hemoglobin of <7.5 g/dL, a fracture of the pubic symphysis, and a transfusion requirement of >2 units per hour of packed red blood cells (PRBC) [119,120]. Multiple lesions with more severe bleeding are found more frequently in patients requiring repeated AGs [121]. Interestingly, our study highlights that the main finding in repeated AGs is represented by the detection of a new bleeding site different from the one identified during the first AG; this finding is consistent with a previous study by Gourlay et al. [119].

Some limitations of the study should be addressed. The main one is represented by the retrospective structure of the analysis. Other limitations are the short follow-up, the heterogeneity of the indications for AG/EA, and the presence of numerous confounding factors such as the coexistence of extra-pelvic injuries and medical conditions arising after the AE.

## 5. Conclusions

In patients undergoing angioembolization for pelvic trauma-related bleeds, the rates of technical success, clinical success, and 30-day bleeding-related mortality were comparable between the group with hemodynamic instability and the group without hemodynamic instability, thus demonstrating that transcatheter arterial embolization is an effective option for the management of traumatic pelvic arterial lesions even in a hemodynamically unstable patient.

Despite technical variations such as the greater use of prophylactic angioembolization in the group with hemodynamic instability, no significant differences in the rate of procedure-related complications were noted. The correction of coagulopathy should not delay TAE or vice versa, as better clinical outcomes were observed if the correction of coagulopathy occurred within 24 h of TAE.

## Figures and Tables

**Table 1:** Population data.

Variables	All Patients (*n* = 116)
Age (years)	56.5 (±23.4)
Sex (M/F)	82 (70.7%)/34 (29.3%)
BMI	26.1 (±4)
eGFR (mL/min)	69.3 (±24.1)
INR	1.3 (±0.3)
aPTT (s)	41.1 (±5.9)
Platelet count (No. ×10^3^/μL)	325.1 (±109)
Coagulopathy (no/yes)	80 (69.0%)/36 (31.0%)
- INR > 1.5	32 (27.6%)
- aPTT > 45 s	32 (27.6%)
- PLT < 80,000/mm^3^	10 (8.6%)
Baseline hemoglobin (g/dL)	7.9 (±0.9)
Antiplatelet therapy	23 (19.8%)
Anticoagulant therapy	35 (30.2%)
Antiplatelet AND anticoagulant therapy	0 (0%)
Antiplatelet OR anticoagulant therapy	58 (50.0%)
Mechanism of pelvic trauma	
- Blunt	102 (87.9%)
- Penetrating	14 (12.1%)
Hemodynamic stability/instability	72 (62.1%)/44 (37.9%)
Young–Burgess classification of pelvic fracture	
- Stable	36 (31.0%)
- Unstable	80 (69.0%)
WSES classification pelvic ring injuries	
- Minor (grade I)	24 (20.7%)
- Moderate (grade II/grade III)	48 (41.4%)–(20.7%/20.7%)
- Severe (grade IV)	44 (37.9%)
Injury Severity Score	28.8 (±15)
Extra-pelvic injury	78 (67.2%)
CT angiography execution	98 (84.5%)
Bleeding on CT angiography	86 (74.1%)
- Direct sign	56 (65.1%)
- Indirect sign	30 (34.9%)
Hematoma volume (mL)	289.1 (±309)

**Table 2:** Procedure data.

Variables	All Patients (*n* = 116)
Bleeding on XA	
- No (blind embolization)	6 (5.2%)
- Yes (targeted embolization)	110 (94.8%)
Site of bleeding	
- Internal iliac artery (uni-/bi-lateral)	104 (89.7%)–(62.1%/27.6%)
- External iliac artery	6 (5.2%)
- Internal AND external iliac arteries	6 (5.2%)
Main bleeding vessel	
- Superior gluteal	36 (31%)
- Iliolumbar	17 (14.6%)
- Lateral sacral	5 (4.3%)
- Inferior gluteal	6 (5.2%)
- Superior vesical	5 (4.3%)
- Inferior vesical/vaginal	4 (3.4%)
- Middle rectal	6 (5.2%)
- Internal pudendal	14 (12.1%)
- Obturator	13 (11.2%)
- Others (e.g., uterine, ext. iliac branches, etc.)	10 (8.7%)
Number of embolized vessels	1.4 (±0.5)
Type of angioembolization	
- Prophylactic (uni-/bi-lateral)	60 (51.7%)
- Distal	56 (48.3%)
Main embolic agent	
- Temporary (gelatin sponge)	66 (56.9%)
- Others	50 (43.1%)
o Coils	30 (25.9%)
o PVA particles or microspheres	2 (1.7%)
o NBCA	10 (8.6%)
o NALEAs (Onyx or Squid)	8 (6.9%)
Intraoperative contrast medium (mL)	36.8 (±15.4)
Volume of contrast to creatinine clearance ratio	0.7 (±0.8)
Vascular access site	
- Femoral	108 (93.1%)
- Radial	4 (3.4%)
- Brachial	4 (3.4%)
Sheath diameter, 4F/5F/6F/≥ 7F	22 (19.0%)/76 (65.5%)/14 (12.1%)/4 (3.4%)
Door-to-groin puncture time (min)	91.2 (±76.2)
Procedure time (min)	30.2 (±10.9)
Time-to-embolization time (min)	120.1 (±75.4)
Fluoroscopy time (min)	9.5 (±4)
Cumulative air kerma (mGy)	169.6 (±66.8)
Dose area product (DAP) (Gy/cm^2^)	27.4 (±10.4)

**Table 3:** Outcomes data.

Variables	All Patients (*n* = 116)
Technical success	116 (100%)
Clinical success	106 (91.4%)
Coagulopathy correction within 24 h of TAE	36 (100%)
Vascular access site hemostasis	
- Manual compression	50 (43.1%)
- Vascular closure device	66 (56.9%)
Units of packed red blood cells transfused per patient	1.5 (±1.5)
Rebleeding	22 (19.0%)
Repeated XA	22 (19.0%)
- Same bleeding site	8 (36.4%)
- Different bleeding site	14 (63.6%)
Non-target embolization	0 (0%)
Trauma-induced coagulopathy (TIC) occurrence after TAE	28 (24.1%)
Procedure-related complication rate	14 (12.1%)
Vascular access site complication (VASC) rate	4 (3.4%)
Procedure-related complications (SIR classification)	
- None	102 (87.9%)
- Minor (grades 1-2)	13 (11.2%)
- Major (grades 3-4-5)	1 (0.9%)
Procedure-related complications (CIRSE classification)	
- None	102 (87.9%)
- Grade 2	6 (5.2%)
- Grade 3	8 (6.9%)
Treatment required for complications	
- None	6 (42.8%)
- Medical	7 (50%)
- Interventional	0 (0%)
- Surgical	1 (7.2%)
30-day bleeding-related mortality	14 (12.1%)
30-day mortality	20 (17.2%)

**Table 4:** Comparison of data between Group 1 (patients with hemodynamic stability) and Group 2 (patients with hemodynamic instability).

Variables	Group 1 (*n* = 72) Hemodynamic Stability	Group 2 (*n* = 44) Hemodynamic Instability	*p*-Value
Age (years)	57.5 (±23)	54.8 (±24.3)	0.3246
BMI	26.1 (±4.2)	26.1 (±3.8)	0.8798
INR	1.33 (±0.3)	1.26 (±0.3)	0.2446
Coagulopathy	22 (30.5%)	14 (31.8%)	1
Baseline hemoglobin (g/dL)	8 (±0.3)	7.7 (±0.8)	0.0869
Young–Burgess classification of pelvic fracture (stable/unstable)	24 (33.3%)/48 (66.7%)	12 (27.3%)/32 (72.7%)	0.5404
Injury Severity Score	27.4 (±15.4)	31 (±14.1)	0.2593
Extra-pelvic injury	46 (63.9%)	32 (72.7%)	0.4156
Hematoma volume (mL)	222.3 (±226.4)	398.5 (±388.5)	0.2704
Prophylactic angioembolization	20 (27%)	40 (90.9%)	<0.0001
Temporary embolic agent (gelatin sponge)	28 (38.9%)	38 (86.4%)	<0.0001
Time-to-embolization time (min)	139.2 (±75.6)	64.6 (±64.6)	<0.0001
Technical success	72 (100%)	44 (100%)	1
Clinical success	66 (91.7%)	40 (90.9%)	1
Coagulopathy correction within 24 h of TAE	22 (100%)	14 (100%)	1
Rebleeding	14 (19.4%)	8 (18.2%)	1
Trauma-induced coagulopathy (TIC) occurrence after TAE	14 (19.4%)	14 (31.8%)	0.1794
Procedure-related complication rate	8 (11.1%)	6 (13.6%)	0.7717
Vascular access site complication (VASC) Rate	2 (2.8%)	2 (4.5%)	0.6335
30-day bleeding-related mortality	8 (11.1%)	6 (13.6%)	0.7717
30-day mortality	10 (13.9%)	10 (22.7%)	0.3108

**Table 5:** Comparison of efficacy and safety outcomes between prophylactic angioembolization and targeted angioembolization.

Variables	Prophylactic Angioembolization (*n* = 60)	Targeted Angioembolization (*n* = 56)	*p*-Value
Technical success	60 (100%)	56 (100%)	1
Clinical success	60 (100%)	46 (82.1%)	0.001
Rebleeding	8 (13.3%)	14 (25%)	0.109
Procedure-related Complication rate	6 (10%)	8 (14.3%)	0.479

## Data Availability

The data presented in this study are available upon request from the corresponding author. The data are not publicly available because of privacy issues.

## Abbreviations

AE: angioembolization; AG: angiography; APTT: activated partial thromboplastin time; ATLS: advanced trauma life support; CID: contrast-induced nephropathy; CKD: chronic kidney disease; CIRSE: Cardiovascular and Interventional Radiological Society of Europe; CTA: CT angiography; DAP: dose–area product; E-FAST: extended focused assessment for sonographic evaluation of trauma patients; eGFR: estimated Glomerular Filtration Rate; EVOH: ethylene–vinyl alcohol; ICU: intensive care unit; INR: international normalized ratio; ISS: Injury Severity Score; LOS: length of stay; NALEA: non-adhesive liquid embolic agent; NBCA: N-butyl cyanoacrylate; PPP: preperitoneal pelvic packing; PRBC: packed red blood cells; PT: prothrombin time; PVA: polyvinyl alcohol; SIR: Society of Interventional Radiology; TAE: transcatheter arterial embolization; TIC: trauma-induced coagulopathy; TTE: time-to-embolization; VASCs: vascular access site complications; WSES: World Society of Emergency Surgery; XA: X-ray angiography.
